# Simultaneous Automated Insect Monitoring Across a Remote Tropical Elevation Gradient With Mothbox

**DOI:** 10.1093/icb/icag072

**Published:** 2026-06-03

**Authors:** Hubert A Szczygieł, Andrew Quitmeyer, Brianna Johns, Daisy H Dent, Yash Sondhi, Daniel Zuleta, Alexandre Antonelli

**Affiliations:** Department of Biological and Environmental Sciences, Gothenburg Global Biodiversity Centre, University of Gothenburg, Medicinaregatan 7B, 413 90 Göteborg, Sweden; Smithsonian Tropical Research Institute; Apartado 0843-03092, Balboa, Ancón, República de Panamá; Digital Naturalism Laboratories, Sibert Ave 123B, 0801, Gamboa, Colón Province, Panama; Department of Human Centered Design & Engineering, University of Washington, 3960 Benton Lane NE 428, Seattle, WA 98195, USA; School of Biological Sciences, Monash University, Clayton, Victoria 3800, Australia; Smithsonian Tropical Research Institute; Apartado 0843-03092, Balboa, Ancón, República de Panamá; Max Planck Institute of Animal Behavior, 78467, Konstanz, Germany; Department of Environmental Systems Science, ETH, 8092, Zürich, Switzerland; Case Western Reserve University, Department of Biology, 10900 Euclid Avenue, Cleveland, OH 44106, USA; Department of Biological and Environmental Sciences, Gothenburg Global Biodiversity Centre, University of Gothenburg, Medicinaregatan 7B, 413 90 Göteborg, Sweden; Department of Biological and Environmental Sciences, Gothenburg Global Biodiversity Centre, University of Gothenburg, Medicinaregatan 7B, 413 90 Göteborg, Sweden; Royal Botanic Gardens, Kew, Richmond, London TW9 3AE, UK; Department of Biology, University of Oxford, South Parks Road, Oxford OX1 3RB, UK

## Abstract

Insect declines have been recorded in many parts of the world, however, the vast majority of taxa and ecosystems, particularly in the tropics, remain poorly documented. Monitoring insects in the tropics is challenging due to their immense diversity, and the limited resources for research. It is therefore crucial to maximally leverage existing scientific capacity. In complement to DNA-based approaches, or as an alternative that bypasses some of their shortcomings, automated, passive insect monitoring devices are a new tool that can assess insect diversity with standardization and scalability. An additional benefit of insect monitoring devices is the ability to program simultaneous, autonomous monitoring in remote regions that would be very resource intensive to monitor with traditional methodologies. Here, we describe the results of an insect monitoring expedition in Cerro Hoya National Park, Panama, which utilized 19 Mothboxes (automated light traps) deployed across an elevation gradient from 119 to 1534 m above sea level. Images were processed by the Mothbot computer vision system and manually validated at order level. For further validation, we sorted one order, – Coleoptera (beetles), to the level of morphospecies. Three days of sampling yielded 64,352 insect detections representing 17 orders. Within the Coleoptera, we detected 26 families and 142 species. Species richness and Shannon diversity decreased with increasing elevation, despite signs of anthropogenic disturbance at lower elevations. The number of detections (a proxy for activity patterns and abundance) also decreased with elevation except for the highest sampling points. Across all elevations, insect activity was greatest at the beginning of the night, with 40% of all insect detections occurring within an hour and a half of sunset, however trends differed between taxonomic groups. This study highlights the potential for automated insect monitors to enable large-scale insect monitoring in remote locations with small teams. Automated insect monitoring does not replace entomologists, but rather greatly expands their capacity for monitoring insect diversity at scale.

## Introduction

Insect declines have been documented in many parts of the world and there is strong consensus that more monitoring is needed to understand where and why these losses are taking place (Dirzo et al. 2014; Sánchez-Bayo and Wyckhuys 2021; Wagner et al. 2021). The tropics contain the greatest concentrations of insect diversity; yet, they remain severely underrepresented in monitoring studies, due to the sheer scale of taxonomic diversity, the logistical constraints of fieldwork, and more limited resources than in the Global North (Simmons et al. 2019; Montgomery et al. 2020; Halsch et al. 2021; Sánchez Herrera et al. 2024).

One answer to the need of expanding insect monitoring capacity is DNA metabarcoding. This is a powerful approach to estimate the diversity of insects in bulk samples, such as Malaise traps, though it remains relatively costly, often requires special permits that grant access to genetic resources, and is often unable to distinguish between species due to the use of taxonomically broad primers and insufficient inter-specific genetic variation. Additionally, DNA metabarcoding requires well-identified reference sequences such as those provided by the Barcode of Life project for identifications (Ritter, Häggqvist, et al. 2019). In complement to DNA-based approaches, or as an alternative that avoids some of their limitations, automated passive insect monitoring devices offer a new tool for assessing insect diversity with standardization and scalability. Compared with metabarcoding, this approach is relatively inexpensive and does not require the collection or handling of specimens. However, the use of automated insect monitoring devices remains constrained by the limited availability of reliably identified reference images, similar to the shortage of reference DNA sequences used in metabarcoding. Producing these reference images, however, is cheaper and less resource-intensive than generating reference sequences. An additional benefit of automated insect monitoring devices is the ability to program simultaneous, autonomous monitoring in remote regions that would be very resource intensive to monitor with other methodologies. New, automated insect monitoring technologies therefore promise to greatly increase global insect monitoring capacity via scalable, standardized data collection (van Klink et al. 2024; Boyle et al. 2025).

A significant challenge in large-scale insect monitoring is that activity patterns are highly sensitive to fluctuating environmental conditions, such as wind, precipitation, lunar phase, and artificial light (Williams 1961; Montgomery et al. 2021). Logistical difficulties in sampling, particularly in landscapes with difficult terrain and harsh sampling environments—typical of much of the tropics—mean that sampling often occurs in asynchronous field campaigns. Measuring and correcting for environmental variables can be difficult, and asynchronous monitoring may obscure biological trends across locations by confounding them with environmental and stochastic variation. Because they are autonomous, automated monitoring technologies can help alleviate this challenge via simultaneous monitoring across sensor networks.

Automated light traps pair an attractant light with a programmable camera, to photograph insects attracted to the light (Bjerge et al. 2021). Computer vision models then crop out and identify the attracted insects (Svenning et al. 2026). The Mothbox, developed by members of our team, is an automated light trap designed in the tropics and optimized for remote deployments.

Here, we present the results of an expedition to Cerro Hoya National Park, Panama, where we deployed 19 Mothboxes to conduct what is, to the best of our knowledge, the first simultaneous automated insect census across a remote tropical elevation gradient. The key objective of this study is to test whether this technology can capture fine-scale ecological patterns, such as activity peaks of different taxa, across a spatial scale that is difficult to sample with traditional methods. Following historical benchmarks (Wolda 1987), we hypothesize that insect richness will decline with increasing elevation, and explore whether modern climatic pressures have shifted these communities compared to 20th-century baselines (Chen et al. 2009).

## Methods

### Field site

**Fig. 1:**
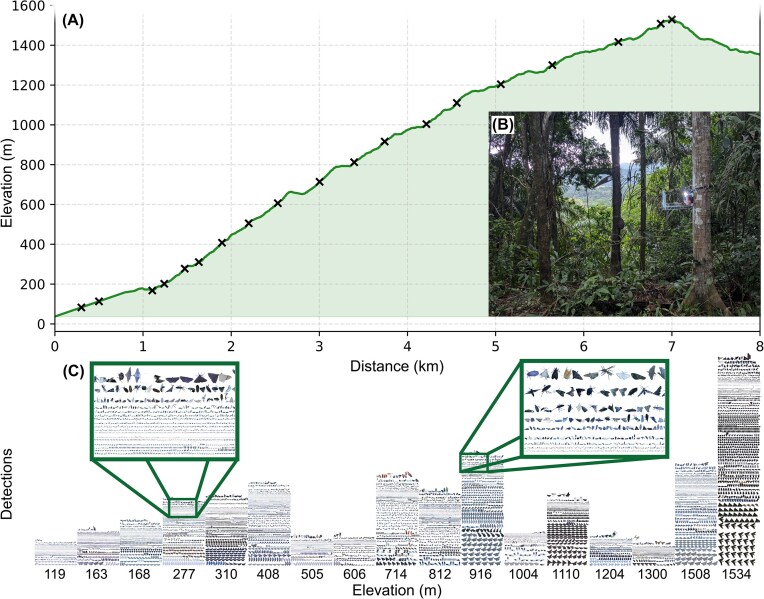
Field site elevation profile and detections. (A) Elevation profile of ridgeline in Cerro Hoya NP, Panama, with Mothbox deployment locations marked with “x.” (B) Mothbox attached to a tree in Cerro Hoya NP. (C) Compiled detection photos for each assessed elevation on one night of the expedition (27 January 2025). Each of the 25,537 insect detections from that night is presented to scale with other insects, showing that communities were dominated by smaller-bodied insects at some elevations on this night. Insets show magnified detections. Of the 19 Mothboxes deployed, two (elevations 202 m and 1416 m) were excluded from analysis due to errors in data collection.

The study site is a ridge on the north side of Cerro Hoya National Park, Panama. Cerro Hoya National Park is an isolated, mountainous region of mature forest that protects habitats from sea level to over 1500 m, representing a sky island extension of Talamancan montane forests (Miller et al. 2015). The lower reaches of the elevation transect are situated in hot, seasonal moist broadleaf forest, while the upper reaches are in a cloud-forest habitat characterized by shorter trees and abundant epiphytes. The sampled ridgeline is very steep, so the lowest elevation point is only 5.2 km away from the highest elevation point (Fig. 1A). The close proximity of sampling points reduces environmental variation unrelated to elevation, thereby limiting the number of confounding covariates that could influence differences in insect communities. Forest cover starts at an elevation of 155 m at the base of the ridge, and the top of the ridge is at 1534 m. The ridge starts in the private land and enters Cerro Hoya National Park at an elevation of 1120 m. The lower reaches of the elevation gradient have seen varying levels of human disturbance, from total forest clearance and partial replanting with non-native timber species at the lowest elevations, to selective logging of valuable hardwoods up to approximately 500 m. Above 500 m, the forest appears to be mature with minimal human impact (Ministerio de Ambiente de Panamá 2021). To our knowledge, this is the first systematic insect monitoring of this elevational gradient.

### Data collection

We conducted our study between 26th and 29th January, 2025, coinciding with a new moon and the start of the dry season. Nineteen Mothboxes were deployed approximately every 100 m of elevational change along the elevation gradient, with extra Mothboxes deployed at the bottom and the top of the gradient. The Mothboxes used were version 4.5 (DIY oriented design), with two internal Mothbeams as attractant lights, one with LEDs in the ultraviolet range and one focused on white light and lower frequencies (Szczygieł et al. 2026). See the Mothbox website for full hardware specifications (https://digital-naturalism-laboratories.github.io/Mothbox/). All Mothboxes were attached to trees with straps 1.5–2 m above the ground (Fig. 1B). The Mothbox at the bottom of the transect, which was situated in a grassy field outside the forest, was equipped with an external solar panel, while the remaining Mothboxes were equipped with additional external batteries. Because insects drawn to light traps tend to stay while the lights are on, our sampling schedule included five discontinuous hours (hereafter “sessions”). This allowed the sheet to clear of insects between sessions, and a new cohort to arrive each time the lights were turned on again. All the Mothboxes used in this study were programmed to run on the same schedule with five discontinuous one-hour sessions per night (19:00–20:00; 21:0–22:00; 23:00–00:00; 02:00–03:00; 04:00–05:00). Sunset was at 18:30 and sunrise was at 6:40 am. The 16 Mothboxes < 1300 m were deployed on the first day of our study and ran for all three nights, while the three Mothboxes located > 1300 m were deployed on the second day of the study and only ran for two nights. Of the 19 Mothboxes deployed, one did not collect data due to a technical failure (elevation 202), another one collected some data, but experienced errors in the field and was excluded from analysis (elevation 1416), and a third collected data as expected and was included in analysis, though parts of some photos were corrupted (elevation 1204). Research permit (ARG-017–2024) for fieldwork in the Republic of Panama provided by Autoridad Nacional del Ambiente.

### Image processing and data validation

All images collected by the Mothboxes were processed with the Mothbot data analysis system. After detections were extracted, we set the identification taxonomic threshold to order level and used a species list generated on GBIF that includes all insects in Panama and Costa Rica, filtered for accepted taxa (DOI 10.15468/dl.8p8wua). The resulting identified detections were clustered perceptually and opened in the Mothbot Classify user interface (https://digital-naturalism laboratories.github.io/Mothbox/docs/processing/classify/classify/). All Mothbot order level identifications were validated and corrected where necessary, then finer level taxonomy and morphospecies were assigned to each detection for Coleoptera. Voucher images can be found on iNaturalist (Mothbox iNaturalist 2025), and identifications were further validated and refined by the iNaturalist community.

### Statistical analyses

We calculated richness and Shannon diversity index using the “Vegan” package (Oksanen et al. 2026) in R v. 4.5.0 (“R: The R Project for Statistical Computing” 2026). We tested the effects of elevation and sampling session on insect activity, richness, and Shannon diversity. Activity was expressed as mean detections per photo (total detections divided by number of photos per site per night, or per site per session) to account for sampling effort. We used generalized linear mixed models to test whether elevation significantly affected (1) mean detections per photo across all insects, and (2) morphospecies richness per site-night. Our model had log(number of photos) as an offset, elevation as a linear predictor, and a random intercept for site to account for repeated sampling nights at each elevation. Poisson and negative binomial families were compared by AIC; the better-fitting family was retained. Shannon diversity was analyzed with a Gaussian linear mixed model: Shannon index ∼ elevation + (1 | site). We assessed assumptions for these three elevation models with DHARMa simulated residuals (uniformity and dispersion tests) (Hartig 2016). For the most frequently detected orders (Lepidoptera, Coleoptera, Hemiptera, and Diptera), we applied the same count mixed-model design to test the effect of elevation on order-specific detections per site-night. We then tested the effect of sampling session on mean detections per photo for each focal order using linear mixed models with session as a fixed factor and random intercepts for site and site-night. Finally, we fitted session × elevation mixed models for each focal order to test main effects of session and elevation and their interaction on mean detections per photo. All analyses were performed in R v. 4.5.0 (“R: The R Project for Statistical Computing” 2026).

## Results

### Mothbox performance

A total of 13,818 source images were taken by the Mothboxes during this study. Mothbot generated a total of 81,818 detections, with 17 orders and 26 Coleoptera families represented across all nights at all elevations. A total of 142 unique Coleoptera species were recognized as unique species/morphospecies, but only 15 of the species could be readily identified to species level by a general entomologist, the remaining 127 (89.4%) were assigned morphospecies names. Of the 81,818 detections, 64,352 were of insects, 91 were of spiders, and 17,411 were errors (for example splotches on the sheet or parts of insects). Mothbot gave correct order level identifications to 61.07% of the insect detections. During validation, 3745 detections were assigned Class-level identification (it was apparent that they were insects, but order level identification was not possible). Order level validation was rapid, requiring a total of ∼30 person-hours for all 81,818 detections. Morphospecies designation took considerably longer, and is ongoing for the other insect orders represented in the dataset.

### Insect activity and elevation patterns

**Fig. 2:**
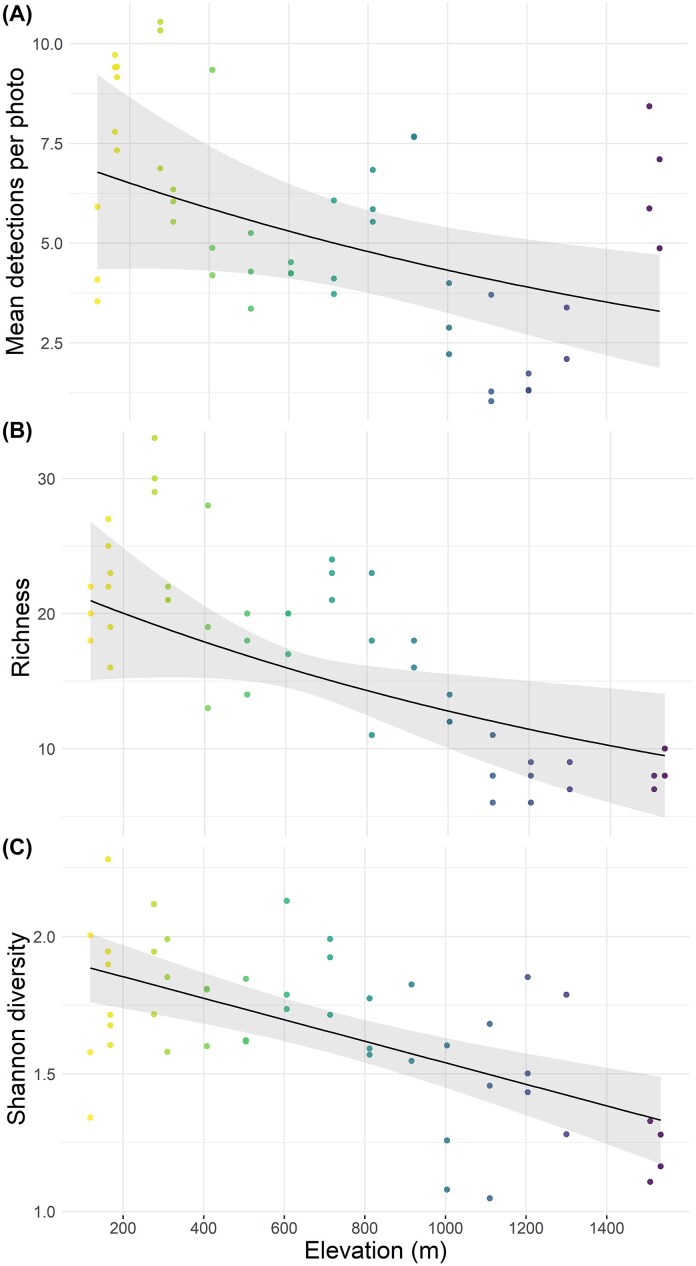
Insect diversity across a tropical elevation gradient. (A) Mean detections per photo across the elevation gradient (B) richness across the elevation gradient (C) Shannon diversity across the elevation gradient, treating number of detections as a proxy for abundance. Points represent site-night values, colored according to elevation; lines and shaded intervals show mixed-model fits. All three variables were inversely correlated with elevation (*P* < 0.05).

**Fig. 3:**
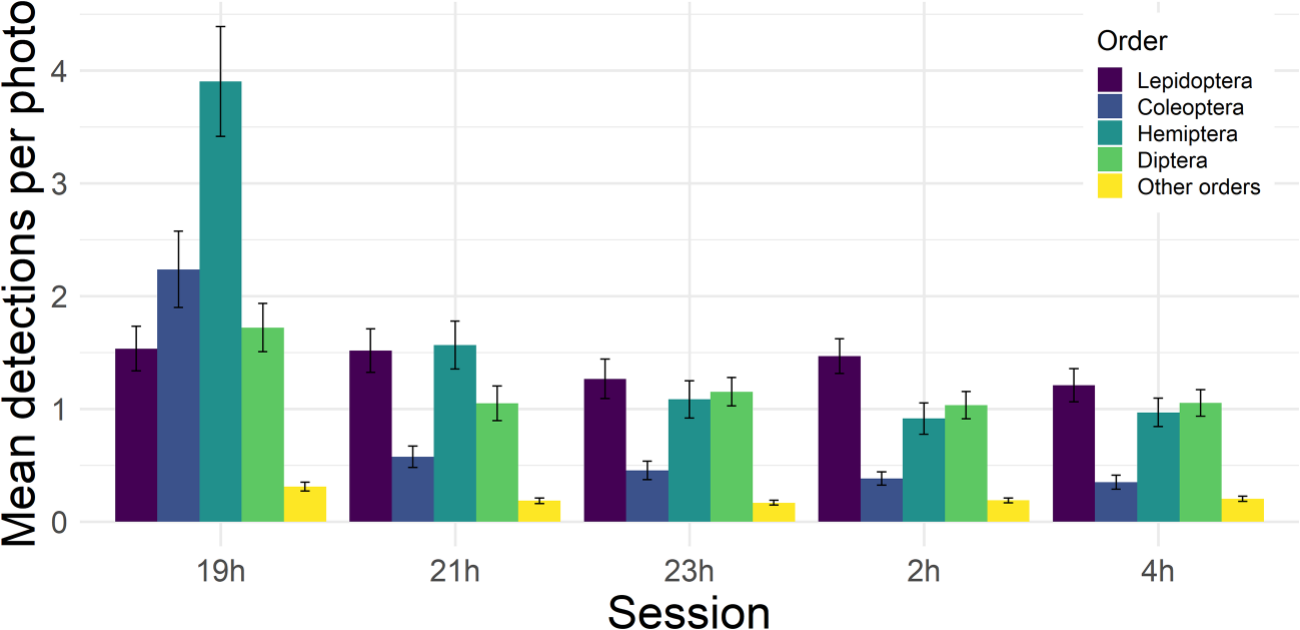
Insect activity and time of night. Mean detections per photo across the five nightly sessions, averaged across all site-nights, separated by Order, for the four most abundant orders and all other orders combined. The sampling intervals were one hour long, starting at each hour indicated on the *x*-axes. Session was a significant predictor of mean detections per photo for Coleoptera, Hemiptera, and Diptera (*P* < 0.05), but not for Lepidoptera. Error bars represent standard error.

**Fig. 4:**
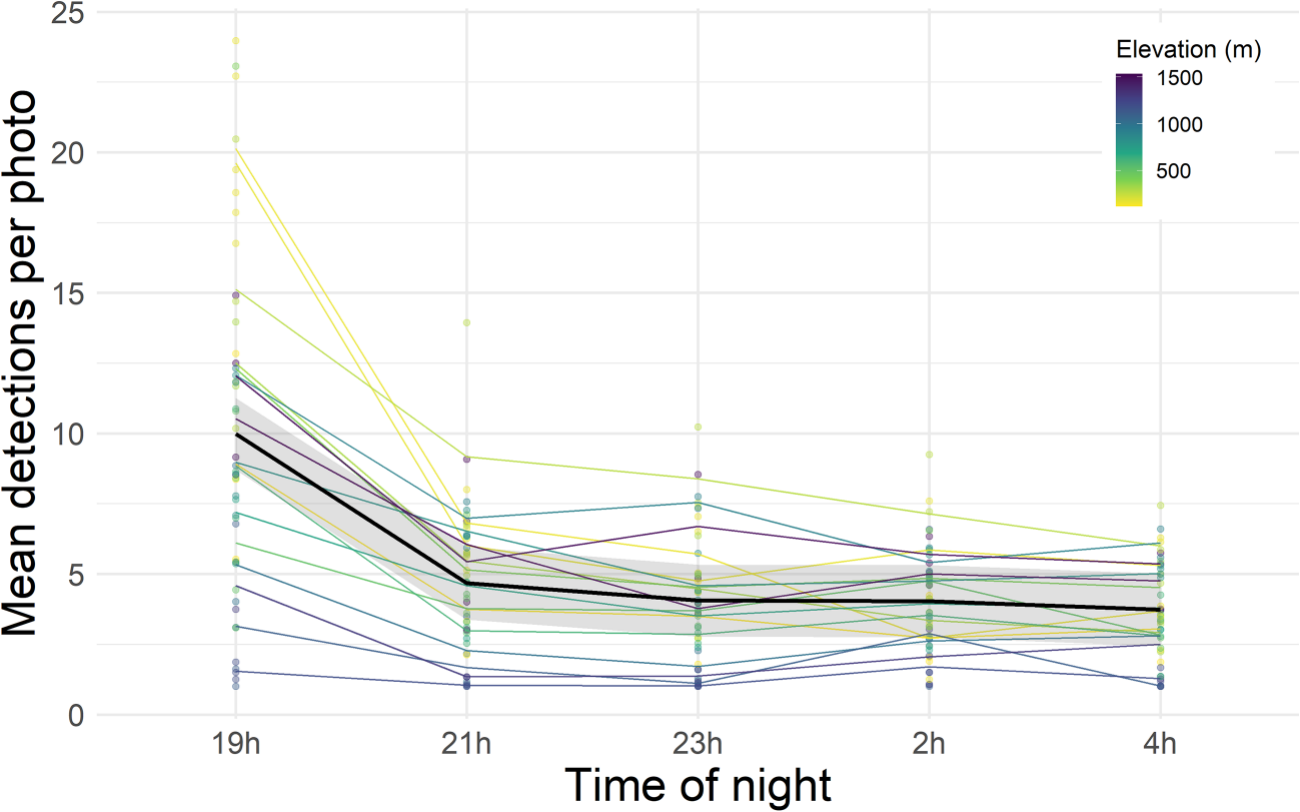
Insect activity and time of night across elevation. Mean detections per photo for all taxa combined, shown for each of five nightly sessions. Each point is one site-night session; color indicates elevation (m). Thin colored lines connect the mean rate at each elevation across sessions. The thick black line shows the overall trend from a linear mixed model (rate ∼ session + random intercepts for site and site-night); the gray band is the 95% confidence interval for the population mean at each session. Sessions were one hour long, starting at the hour indicated on the *x*-axis.

**Fig. 5:**
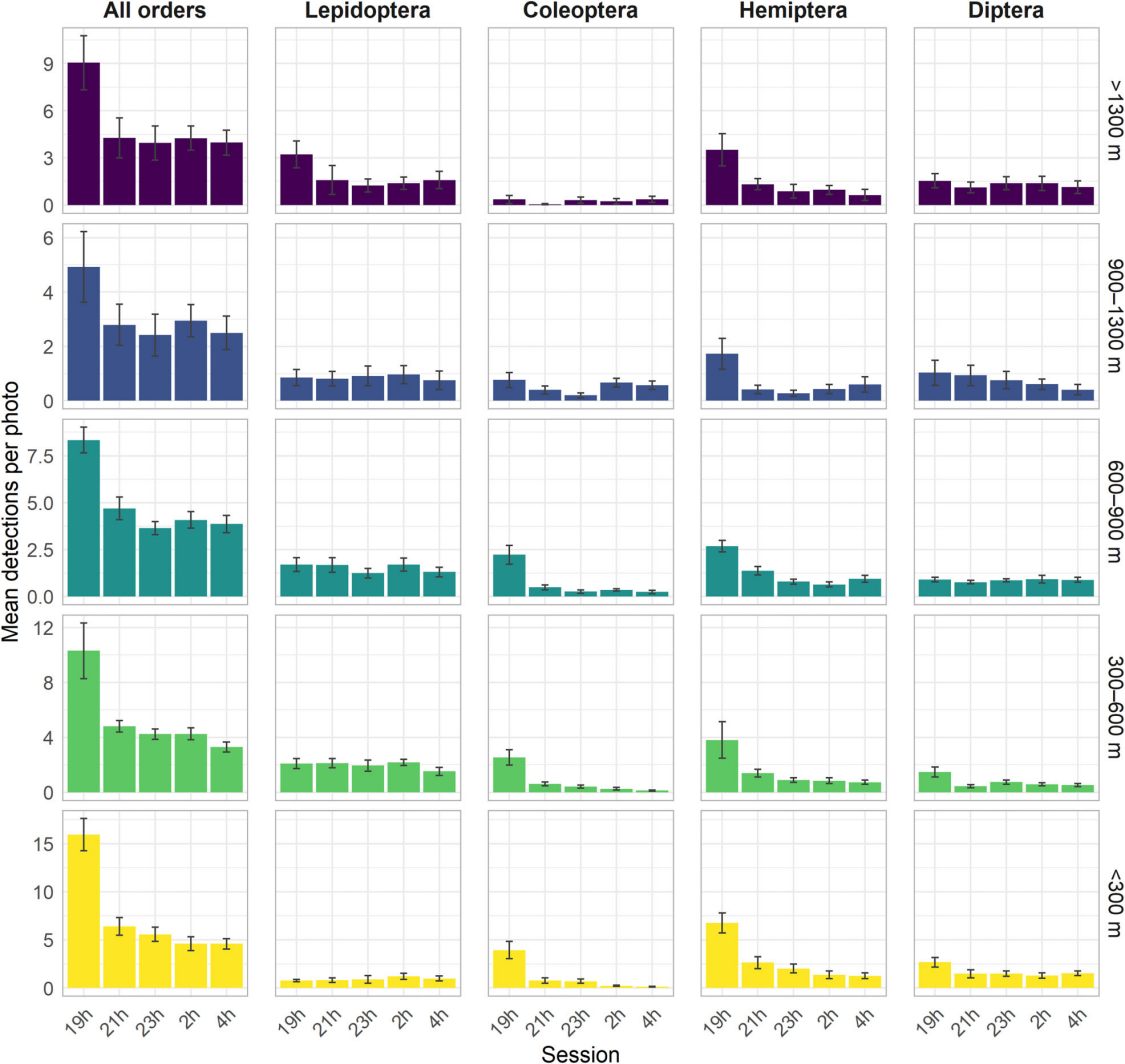
Insect activity by order across time of night and elevation. Mean detections per photo, per session, split by elevation band and taxonomic group. The left column of panels includes data from all 17 orders documented in the study, the remaining columns show results for the four most frequently detected orders. The sessions were one hour long, starting at each hour indicated on the *x*-axes. Session, elevation band, and the interaction of these variables was a significant predictor of mean detections per photo for Coleoptera and Hemiptera, sampling session (hour), elevation, and their interaction were significant predictors of mean detections per photo (mixed models with continuous elevation and random effects for site and site-night; all *P* < 0.05). For Diptera, session, elevation, and the session × elevation interaction were also significant (*P* < 0.05). For Lepidoptera, only the session × elevation interaction was significant (*P* = 0.012); neither session alone (*P* = 0.19) nor elevation alone (*P* = 0.052) reached significance at α = 0.05. Error bars represent standard error.

Insect detection rate, richness, and Shannon diversity decreased with increasing elevation (*P* < 0.05; Fig. 2). The two sampling points at the top of the ridge (over 1500 m elevation) had much higher activity values than the next highest points. Insect activity was greatest during the first session each night (19:00–20:00), a pattern that was consistent across nearly all elevations (Figs. 3 and 4). Orders of insects differed in their nocturnal activity patterns, with Coleoptera and Hemiptera particularly active in the early evening. Lepidoptera, on the other hand, had fairly consistent activity throughout the night (Fig. 3). However, these patterns differed across the elevation gradient (Fig. 5). Lepidoptera were most active during the first sampling session only at higher elevations, while the converse was true for Coleoptera.

## Discussion

### Activity and elevation patterns

Most insects are active at night (Wong and Didham 2024), however relatively little is known about nocturnal insect activity patterns and how they vary among taxa (Hutchins 1940; Graham et al. 1964; Charvalakis et al. 2026; Köhler et al. 2026). In this study, we found that insect activity was greatest during the first session each night (between 19:00 and 20:00), a pattern that held across most elevations and taxa (Figs. 3, 4, and 5). This is consistent with a study which used an early version of an automated light trap and found a generally decreasing trend in abundance throughout the night (Hutchins 1940), however it stands in contrast to two European studies which show activity peaks around the middle of the night (Charvalakis et al. 2026; Köhler et al. 2026). Our study shows that future light trapping of Lepidoptera should likely include sampling throughout the night, as Lepidoptera activity overall did not peak at the start of the night as it did for other orders (Fig. 3), except at higher elevations (Fig. 5). The early activity peak observed for most taxa could be the result of mostly-diurnal taxa, which are still active after sunset while the environment retains some heat of the day. The difference between our results and those from northern latitudes could be the result of tropical ectotherms having narrower thermal optima, thus being more sensitive to temperature fluctuations throughout the day (Deutsch et al. 2008). Ultimately, more studies of nocturnal activity patterns using automated light traps like the Mothbox, particularly paired with simultaneous monitoring of environmental conditions, will shed more light on insect behavioral ecology.

Declines in insect richness and diversity with elevation observed in our study was consistent with findings from a study conducted 40 years previously that used light traps to survey insects at multiple locations in Panama spanning an elevation gradient (Wolda 1987). A global metanalysis of insect richness across elevation gradients shows a low-elevation plateau with decreasing richness (Dolson and Kharouba 2024). The decline in richness observed in our study (Fig. 2B) does not follow this trend, it appears to decrease linearly. Cloud forest specialists have started to decline as their habitat has shrunk due to climate change in other parts of the American tropics (Ponce-Reyes et al. 2012; Helmer et al. 2019), but it is difficult to say if this occurred in Cerro Hoya, as there are no historical baseline data for comparison.

### Mothbox performance

Conducting a simultaneous sampling campaign across the elevation gradient in this study would have been far more resource intensive using traditional monitoring methods, for example using bucket traps that are left overnight and processed in the morning. The fieldwork logistics and human-power required to operate traps across the entire elevational gradient for three nights would render the work impractical for most research teams. Furthermore, using traps to monitor whole insect communities as opposed to particular target taxa would also require months of manual sample processing, assuming the same sampling intensity used here. Critically, collecting comparable temporal data with traditional methods would also demand substantial effort and continuous overnight sampling (Köhler et al. 2026), which was not feasible at the study site. Manual sorting and identification of tens of thousands of insects, even to order level, would have required months of work rather than the week needed for initial validation. Molecular approaches like DNA metabarcoding are sometimes suggested as a method for shortening this timeline (Chua et al. 2023), however in comparison to automated insect monitors this approach is still quite expensive and time consuming, largely due to physical sample collection. In the context of large-scale biodiversity monitoring, we propose that automated insect monitors like the Mothbox are used as a first step to identify areas where investment in sample collection and developing DNA reference libraries would be most valuable. Finally, taxonomy is a declining field, particularly underfunded in the tropics (Slade and Ong 2023; Simões et al. 2026). Automated insect monitoring offers a method for better leveraging existing taxonomic capacity, and the ease of use and low cost of Mothbox will offer an entry point for more local scientists to gain experience with tracking and identifying insects.

A central issue in the analysis of automated light trap data is the interpretation of detections as a proxy for abundance. It is generally accepted that most insect monitoring methodologies do not directly measure population abundance, and instead are a measure of activity rates (Didham et al. 2020). To be trapped, an insect must first be active (inactive insects do not get trapped), and very active insects get trapped more frequently or more easily. Automated light trap detections (timestamped instances of an insect photographed on the target sheet) are similarly not a true measure of abundance. If an insect lands on the sheet and stays there for the duration of several photos, the same insect will be represented in several detections (Fig. 1C), thus detections can exaggerate true abundance. One way to approximate abundance is to use temporal “tracks” of the same insect, and treat each track as a single occurrence record (Hogeweg et al. 2019; Bjerge et al. 2021). Another method (the one employed in this study), is to calculate detections per photo (Möglich et al. 2023). If the number of detections in one photo is more than another, one can assume that the observed abundance at that particular point in time is higher. By using mean detections per photo it is possible to compare activity patterns through time, and to standardize sampling effort (deployments of different numbers of nights, and those with different timelapse intervals). However, not all insects attracted to the sheet actually land on the surface that is being photographed, which results in undercounting relative to lethal trapping methodologies (Möglich et al. 2023). In practice, both detections and tracks are closer to an approximation of insect activity than abundance. It has long been known that light trap catches are highly dependent on insect activity (Williams 1961). Insects that are more active are more likely to be drawn to the attractant lights and be photographed. If insects are more active during a particular time of night, season, or weather conditions, they will be captured in more detections than times of low activity, even if true abundance stays the same.

A final point to consider is the relationship between detections and biomass. It is possible to infer approximate insect biomass from images or abundance records (Ärje et al. 2020; Kinsella et al. 2020). We propose that one quick way to do this with automated light trap detections is via pixel area. The size in pixels of a detection roughly corresponds to the size of the insect detected, and consequently should correspond to its mass. This method of estimation could be further improved with form-fitting segmentation that isolates insects from their background.

The performance of the Mothbot computer vision indicates there is room for improvement; 21.2% of detections were errors that should have been ignored, and, of detected insects, 38.9% were misidentified at Order level. The likely reasons for this are poor resolution of small insects and limited reference imagery. We did not set a lower size threshold for identifications, and the smallest insects, which constituted a significant share of detections, were quite low resolution. Furthermore, existing image reference libraries for insects do not have sufficient coverage in the tropics and are poorly suited to the task of identifying insect detections from automated light traps. The TreeOfLife-200M dataset, which forms the foundation of the latest BIOCLIP model used by Mothbot, is largely composed of digitized museum specimens and in-situ photos, generally of high-resolution (Gu et al. 2025). It is not surprising that identification accuracy was low for our detections of insects with natural wing positions, but on consistent white backgrounds, as models perform more robustly when training data reflects the characteristics of real-world inputs (Ramírez-Agudelo et al. 2025). However, Mothbot Classify makes human validation and identification of pre-sorted detections rapid, creating dependable datasets, and laying the groundwork for training datasets in the future which are based on millions of human-validated automated insect detections.

## Conclusions and future directions

With automated insect monitoring technology, insects have an increased potential to be used as important biodiversity indicators. Plant and bird diversity, which have been traditionally easier to measure at scale, have been shown to be indicative of insect diversity (Schulze et al. 2004; Basset et al. 2012; Zhang et al. 2016). With the rise of automated insect monitoring, the tables may be turning with insects offering more granular and continuous biodiversity data. However, correlations between automated insect detections and other taxonomic groups need to be tested, as there are scale-dependent inconsistencies between diversity patterns of different taxa (Ritter, Faurby, et al. 2019). Due to their diversity, abundance, and sensitivity to environmental conditions, insects can be used as indicators of environmental change (Landmann et al. 2023), and incorporating automated insect monitoring could be a cost-effective way of giving biodiversity greater representation in large-scale monitoring programs like ForestGEO (Anderson-Teixeira et al. 2015). An additional key use of Mothboxes, as demonstrated in this study, will be as a first step in monitoring remote regions, to determine where resources are best directed for field collections. The nascent field of automated insect monitoring has the potential to radically improve how we track biodiversity, and will give insects greater visibility in conservation decisions.

## Data Availability

Data and analysis scripts are available on github: https://github.com/Hubertszcz/Mothbox-Cerro-Hoya Selected insect detections from the Mothbox Cerro Hoya expedition can be found on iNaturalist: https://www.inaturalist.org/observations?nelat=7.37699114571884&nelng=-80.72012927325186&project_id=253230&subview=map&swlat=7.2833490053151175&swlng=-80.80887820513662&verifiable=any

## Notes

From the symposium “Behavioral and environmental monitoring with open-source and low-cost tools” presented at the annual meeting of the Society for Integrative and Comparative Biology, January 3-7th , 2026.
